# Rethinking mental wellness among adolescents: an integrative review protocol of mental health components

**DOI:** 10.1186/s13643-022-01961-0

**Published:** 2022-05-02

**Authors:** Zaida Orth, Brian van Wyk

**Affiliations:** School of Public Health, University of the Western Cape, Bellville, 7535 South Africa

**Keywords:** Adolescent mental health, Mental wellness, Integrative review, Positive psychology

## Abstract

**Background:**

Adolescents have been overlooked in global public health initiatives as this period is generally considered to be the healthiest in an individual’s life course. However, the growth of the global adolescent population and their changing health profiles have called attention to the diverse health needs of adolescents. The increased attention toward adolescent health has accentuated existing gaps as global health reports have emphasised that there is a continued need for valid and reliable health data. In this context, evidence has shown that mental health issues constitute one of the greatest burdens of disease for adolescents. This integrative review aims to unpack the meaning of mental wellness among adolescents and its associated constructs by analysing and synthesising empirical and theoretical research on adolescent mental wellness. In doing this, we will develop a working definition of adolescent mental wellness that can be used to develop an instrument aimed at measuring adolescent mental wellness.

**Methods:**

The integrative review is guided by the five steps described by Whittemore and Knafl. A comprehensive search strategy which will include carefully selected terms that correspond to the domains of interest (positive mental health/mental wellness) will be used to search for relevant literature on electronic databases, grey literature and government or non-governmental organisations (NGO) websites. Studies will be included if they describe and/or define general mental wellness in adolescent populations aged 10–19. The screening and reporting of the review will be conducted following the Preferred Reporting Items for Systematic Reviews and Meta-Analyses (PRISMA) guidelines. Data from the integrative review will be analysed using narrative framework synthesis for qualitative and quantitative studies.

**Discussion:**

This integrative review aims to search for and synthesise current research regarding adolescent mental wellness to identify how wellness is being described and conceptualised. We aim to identify gaps and to contribute to a more comprehensive definition of mental wellness which can aid in the development of an age- and culturally appropriate measure of adolescent mental wellness.

**Supplementary Information:**

The online version contains supplementary material available at 10.1186/s13643-022-01961-0.

## Background

In 2016, adolescents (10–19 years) were estimated at 1.2 billion (18%) of the world population, making them the largest population of adolescents in history [1, 2]. Adolescents have been largely overlooked in global health and social policies, because this period is generally considered to be the healthiest in an individual’s life-course [2], and the unique health problems associated with adolescence have been misconstrued or ignored in favour of more pressing public health concerns [3]. However, changing health profiles of adolescents in both developed and developing countries have called attention to the diverse health needs of adolescents [1]. According to the World Health Organization (WHO), more than 1.1 million adolescents died in 2016—mostly from preventable or treatable causes [4]. Therefore, the considerable gains from global investments in child and maternal health programmes would yield fewer long term benefits without simultaneous investments in adolescent health [5, 6].

According to the life course approach, all stages of an individual’s life are intricately intertwined and interconnected with each other, and other people in society including past, present and future generations of their families [7]. In other words, evidence has shown that early life experiences, including events experienced in the  pre-conception phase, play a role in determining the developmental origins and trajectories of health and wellness or disease across an individual’s life course [7]. From this perspective, it is understood that the health and wellness of individuals, as well as communities, depend on interactions oscillating between multiple risk and protective factors throughout one’s life. Based on this, early and appropriate interventions during child and adolescent years are shown to be the most effective prevention strategies to promote optimal public health and human development [5, 6, 8]. Additionally, following the life course approach, it is argued that these early investments in child and adolescent health will yield a triple dividend as they will grow into healthier adults who can contribute positively to society, as well as the health and development of the next generation [2, 9].

Globally, mental health issues constitute one of the greatest burdens of disease for adolescents. According to the WHO, in 2016 mental health conditions accounted for 16% of the global burden of disease and injury for adolescents, with depression being identified as one of the leading causes of illness and disability, followed by suicide as the second leading cause of death in adolescents and self-harm the third [4, 9]. UNICEF propagates that half of all lifetime mental disorders have onset during adolescence [10]. The recent inclusion of adolescents on the global health agenda as a target group for intervention represents a key step toward reducing the global burden of disease attributed to mental health disorders and reducing preventable deaths [2, 10–13]. However, due to the previous neglect of mental health as a public health issue, efforts to address adolescent mental health are met with various challenges.

Currently, there is a lack of data concerning mental health conditions among adolescents, especially those living in low- and middle-income countries (LMICs) [11]. The lack of a body of quality evidence can affect the way adolescents are represented in national policies, as well as the ways in which government and healthcare officials respond to treatment and prevention [2, 14]. According to WHO, a 2014 review of health policy documents from 109 countries showed that 84% have given some attention to adolescents, with three-quarters of them addressing sexual and reproductive health; one-third addressing tobacco and alcohol use, and one-quarter focusing on mental health [1]. In LMICs, efforts to improve child and adolescent mental health services (CAMHS) are hindered by a lack of specific CAMHS policies, resources and, fewer child and adolescent psychiatrists and other mental health professionals [12, 13]. Furthermore, studies from developed countries have suggested that while CAMHS and policies are in place, there is a lack of mental health service uptake among children and adolescents due to various attitudinal, stigma-related, and structural barriers to accessing mental health services [14]. These challenges and barriers to CAMHS in both LMICs and higher-income countries are particularly apparent among adolescents living with a chronic disorder or disease [15, 16]. Mental health conditions are increasingly recognised in children and adolescents with chronic disorders. Studies have shown that living with a chronic health condition is associated with increased risk of developing comorbid physical and mental health problems, which in turn influence treatment adherence and quality of life [16–18].

Another recurring obstacle for integrating mental health into global public health initiatives and frameworks is the lack of consensus of a definition of mental health [19]. Despite the growth of mental health and wellness research in recent decades, the question of how mental wellness should be defined remains largely unresolved [20]. This has given rise to broad and ambiguous definitions which, consequently, result in concepts such as mental health, mental wellness and mental wellbeing being used interchangeably. Currently, the term ‘mental health’ is often used as a euphemism to refer to mental illness, referring to conditions that adversely affect cognition, emotion and behaviour (i.e. depression and anxiety) [21]. This use reflects in the literature as the majority of adolescent mental health research adopts the dominant pathological view of health by focusing on mental health *disorders* such as psychiatric disorders, general mental health disorders, emotional and behavioural problems and psychological distress [22, 23]. Similarly, global health initiatives such as AAH-HA! focus majorly on the burden of disease of mental disorders by reporting on self-harm, depressive disorders, childhood behavioural disorders and anxiety [3]. This dominant pathological view of mental health persists despite the contributions of positive health and wellbeing research which argues that *wellness* and *illness* are not two ends of the same continuum as previously thought, rather these constructs represent two independent continua [19, 23]. In other words, the absence of mental illness does not necessarily indicate a state of mental health/wellness [1, 23, 24]. Therefore, it is imperative to consider both mental wellness and mental illness in research, and to move away from the previous ‘absence of disease’ model to one that emphasises positive psychological functioning for mental health [23, 24]. In this model, wellness refers to the degree one feels positive about life, and one’s capacity to manage one’s feelings, behaviours, and limitations [23]. From this model, addressing adolescent mental wellness is seen as more than treating and mitigating the burden of disease of mental illness, rather it is also useful in maintaining lifelong mental and physical wellness and preventing the development of mental disorders [23]. Adolescents in particular experience multiple physical, social, and emotional changes, which can positively or negatively impact on their mental wellness. Therefore, interventions at this stage are crucial as research shows that providing psychosocial support and mental health promotion, such as psycho-education and community empowerment, facilitates the development of mental wellness (positive mental health) which is protective against psychopathology (mental illness) [9, 24].

There is a need to develop accurate and culturally appropriate measures of mental wellness to support research endeavours that aim to improve adolescent mental health. Therefore, there is a greater necessity to clarify *what* is being measured, and how the resulting data from the measure should be interpreted to undertake fair and valid assessments. As such, developing a definition of mental wellness should encompass more than the description of wellness itself (as is the case with current definitions) to a clear and definite statement of the exact meaning of the construct.

To this end, this integrative review forms part of a larger study which aims to unpack the meaning of mental wellness among adolescents and its associated constructs by analysing and synthesising relevant literature and empirical and theoretical research on adolescent mental wellness. In doing this, we aim to use this information to develop and conceptualise adolescent mental wellness as a construct. Additionally, by focusing on conceptualising mental wellness, we hope to provide clarity regarding the way concepts such as mental wellness are used in the literature by clearly distinguishing between mental health (as a euphemism for mental illness) and mental wellness as a positive state of mental health. We aim to develop an instrument which can measure mental wellness as an indicator of general mental health and wellness among adolescents.

## Methodology

The integrative review has been identified as a unique tool in healthcare for synthesising investigations available on a given topic or phenomena and for directing practise based on scientific knowledge [25]. The existing body of literature on mental health among adolescents is varied and complex as there are many concepts associated with mental health research ranging from positive aspects such as ‘resilience’ and ‘self-efficacy’ to negative aspects such as ‘depression’ and ‘anxiety’. As such, it is not possible for one study to capture all the dimensions associated with mental health. However, by adopting the integrative review method, we will be able to include the various sources and methodologies used in research to summarise existing empirical and theoretical literature associated with [positive] mental wellness concepts to better understand and conceptualise mental wellness among adolescents. The integrative review method proposed by Whittemore and Knafl [26] will be used: (1) problem identification, (2) literature search, (3) data evaluation, (4) data analysis, and (5) presentation of the integrative review.

### Problem identification

The problem identification stage is a crucial first step in an integrative review. Therefore, we aim to approach this as a phase in itself. This means, going beyond the initial research questions to fully develop a framework of the problem and all its related variables. In this section, we describe some approaches we will use to identify the problem which the integrative review will address. As previously mentioned, our interest lies in understanding how mental wellness is conceptualised among adolescents, to aid in the conceptualisation and development of a mental wellness instrument for adolescent populations. Based on our initial reading of the literature, we have identified two recurring issues in this regard: firstly, there is a lack of validated mental wellness instruments for adolescents; and secondly, despite a growing body of research, the question of how mental wellness should be defined remains largely unresolved. Based on this, we have proposed to follow two research questions to aid us in identifying the problem.

1. How is the concept of mental wellness defined in research involving adolescents?
2. What indicators of mental wellness are being explored/investigated in research?

These two questions allow us to investigate how research has approached the study of mental wellness, what variables were of interest and how these were defined. To answer these questions, we will follow an iterative approach to gather and assess the available information to present a clear identification of the problem and all the variables of interest. To this end, we are currently conducting a systematic review of mental health instruments used in research with adolescent populations [21].

**Fig. 1:**
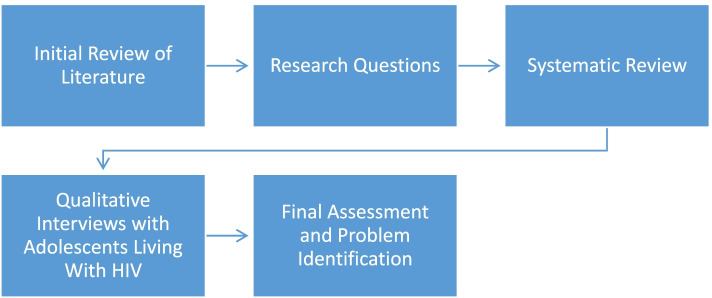
Steps followed to identify the problem for an integrative review

Understanding how mental wellness has been defined in research is an important part of our problem identification, as it will show us what theories and/or definitions of mental wellness are dominant, and which are missing. As Dodge et al. [20] argued, current definitions of wellness are more descriptive in the sense that they describe aspects of wellness rather than the construct itself. This lack of definition poses a problem in measurement development as the definition of a construct ultimately influences how it is being measured and how the resulting data should be interpreted. Therefore, to further aid our problem identification, we will compare the data from the systematic review with data from qualitative interviews exploring mental wellness among adolescents living with HIV (ALHIV). As previously mentioned, this review forms part of a larger study aimed at developing an instrument to measure mental wellness among adolescents. We have chosen to include the interviews with ALHIV for the problem identification stage as we want to develop an instrument that can measure mental wellness among healthy populations and those living with a chronic illness such as HIV. This is necessary as Manderscheid et al. [23] argue that a dual emphasis on mental and physical health is essential as studies have shown that positive health may influence biological functioning. This information will be used to identify the problem of the integrative review (Fig. 1). Using the information from the problem identification phase, we will move on to the second phase to conduct a literature search of mental health concepts used in research with adolescent populations.

### Literature search

A comprehensive search strategy which will include carefully selected terms that correspond to the domains of interest (mental health/mental wellness) will be used to search for relevant literature on electronic databases, grey literature and government or non-governmental organisations (NGO) websites. A systematic database search will be performed using Ebscohost (Psycharticles, Academic Search Premier, SocIndex), Educational Resource Information Center (ERIC), Medical Literature Analysis Retrieval System Online (MEDLINE) and Sabinet. A list of initial keywords has been identified for the search strategy: ((adolescen* OR teenage* OR young people OR youth) [AND] (“psychological wellbeing” OR “mental health wellbeing” OR “mental wellness” OR “mental health”). As the integrative review allows for a more iterative process, the list of keywords will be modified as the initial search reveals more relevant and refined search terms.

### Inclusion and exclusion criteria

Studies will be included if they describe and/or define mental wellness in adolescent populations. As the interest lies in conceptualising mental wellness for adolescents, only studies dealing with general mental health, wellbeing and wellness will be included. In other words, studies focused on mental disorders or mental illnesses among adolescents will be excluded. For this review, studies will be included for all adolescents aged 10–19 who have not been diagnosed with a mental illness or disorder. Eligible studies will include qualitative, quantitative, and mixed-method studies published from 2000 to 2022. The time period of the search strategy was chosen due to the paucity of research in this area [3, 22, 27]. Furthermore, the prioritization of adolescent health and the focus on adolescent-friendly services occurred after 2000 [28].

### Screening and selection process

#### Study selection

The PICOT mnemonics (Table 1) for reviews will be used to guide study selection.

**Table 1:** PICOT based inclusion criteria for literature review

Patient population	Adolescents aged 10–19 years
Intervention or Interest	Definitions of mental wellness or psychological wellbeing or general mental health among adolescents
Comparison	Not applicable
Outcomes	Mental wellness, psychological wellbeing, or general mental health
Time	2000–2022

The above-mentioned criteria and search strategy will be used to search the databases. The screening and reporting of the review will be conducted following the Preferred Reporting Items for Systematic Reviews and Meta-Analyses (PRISMA) guideline and checklist [29]. The number of hits for each database will be recorded and the citations will be exported to Mendeley citation software. Following this, two reviewers will screen all the titles and abstracts to assess which articles are appropriate for inclusion. The full-text articles of the included abstracts will be downloaded and reviewed again to determine which articles should be included for the final assessment [30]. Any discrepancies between the two reviewers will be resolved by a third party. Additionally, based on the information retrieved from the screening, the researcher may modify the search to include other relevant sources.

### Data evaluation

In this integrative review, the primary sources will include both empirical and theoretical literature - which increases the complexity of evaluating the quality of the included sources [26]. According to Whittmore and Knafl [26], integrative reviews using diverse sampling frames may adopt an approach to data evaluation that is similarly used in historical research. In this case, the authenticity, methodological quality, informational value, and representativeness of the available sources should be discussed in the final report. To minimise bias, the two reviewers will utilise two existing quality criteria instruments to evaluate the different types of data [26]. Firstly, the Mixed Methods Appraisal Tool (MMAT) (Additional file 1: Appendix A) will be used to assess the methodological quality of the studies as it allows for summarising the overall quality across a range of study designs [31]. Secondly, the SFS scoring system version E (Additional file 1: Appendix B) will be used to assess the quality of the methodologies of the included articles [32]. The SFS scoring system version E is appropriate as it allows for screening of both quantitative and qualitative research and allows for the appraisal of the definitions of constructs being investigated [32].

### Data analysis

Once the selection of included articles has been finalised, we will extract the relevant data into a Microsoft Excel document to organise the information and prepare for the data synthesis. The Excel sheet will include information regarding the purpose of the study, study characteristics, results, and appraisal of the study as well as any other supporting information. All data will be cross-checked for quality purposes.

Data from the integrative review will be analysed using narrative framework synthesis for qualitative and quantitative studies. Framework synthesis begins with a tentative framework that can either be borrowed from previous studies or can be developed from key concepts [26, 33]. With framework synthesis, the included studies are coded according to the developing framework in an iterative process until the body of evidence can be presented coherently.

In the final stage, the findings from the review will be discussed and presented in either tabular or diagrammatic form. Additionally, the limitations of the review will be discussed as well as recommendations for future research.

## Discussion

This integrative review aims to synthesise current literature on adolescent mental wellness to identify the ways in which this is being described and applied in research. The purpose of this is to identify gaps and to contribute to the conceptualisation of a more comprehensive definition of mental wellness which can aid in the development of an age- and culturally appropriate measure of adolescent mental wellness. Such measures are much needed in adolescent health research as it may be used to better understand the mental wellness needs of adolescents and contribute to the development of interventions and programmes aimed at improving psychological wellbeing and/or mental wellness of adolescents.

## Strengths and limitations

According to our knowledge, this protocol describes the first integrative review to investigate and describe how mental wellness is defined in research among adolescents. Understanding how mental wellness among adolescents has been conceptualised is necessary to identify what are the strengths and limitations of such definitions. This will allow researchers to rethink what mental wellness means to adolescents and how this can and should be measured in research. A limitation of this study is related to the search strategy, notably around the time span (2000–2022) and the identification of grey literature, as not all possible sources of literature may be accessed.

## Supplementary Information

**Additional file 1: Appendix A.** Mixed Methods Appraisal Tool (MMAT). **Appendix B.** SFS Scoring System (Version E). **Appendix C.** PRISMA-P Checklist.

## Data Availability

Not applicable.

## Abbreviations

ALHIV: Adolescents living with HIV
ART: Antiretroviral therapy
CAMH: Child and Adolescent Mental Health
LMICs: Low- and middle-income countries
MMAT: Mixed Methods Appraisal Tool
SFS: SFS Scoring System
UNICEF: United Nations International Children’s Emergency Fund
WHO: World Health Organization
