# Supplementary material for: Rethinking mental wellness among adolescents: an integrative review protocol of mental health components
Source: Syst Rev. 2022 May 2;11:83. doi: 10.1186/s13643-022-01961-0 (PMC9063294; doi:10.1186/s13643-022-01961-0)
Supplement: Supplementary file 1 — Additional file 1: Appendix A. Mixed Methods Appraisal Tool (MMAT). Appendix B. SFS Scoring System (Version E). Appendix C. PRISMA-P Checklist. [file 13643_2022_1961_MOESM1_ESM.docx]

**Appendix A: Mixed Methods Appraisal Tool (MMAT)**

**PART I. MMAT criteria & one-page template (to be included in appraisal forms)**

**Types of mixed methods**

**study components or**

**primary studies**

**Methodological quality criteria (see tutorial for definitions and examples) Responses**

Yes No Can’t

tell

Comments

**Screening questions**

**(for all types)**

 Are there clear qualitative and quantitative research questions (or objectives*), or a clear mixed methods question (or objective*)?

 Do the collected data allow address the research question (objective)? E.g., consider whether the follow-up period is long enough for the

outcome to occur (for longitudinal studies or study components).

***Further appraisal may be not feasible or appropriate when the answer is ‘No’ or ‘Can’t tell’ to one or both screening questions.***

**1. Qualitative** 1.1. Are the sources of qualitative data (archives, documents, informants, observations) relevant to address the research question

(objective)?

1.2. Is the process for analyzing qualitative data relevant to address the research question (objective)?

1.3. Is appropriate consideration given to how findings relate to the context, e.g., the setting, in which the data were collected?

1.4. Is appropriate consideration given to how findings relate to researchers’ influence, e.g., through their interactions with participants?

**2. Quantitative**

**randomized controlled**

**(trials)**

2.1. Is there a clear description of the randomization (or an appropriate sequence generation)?

2.2. Is there a clear description of the allocation concealment (or blinding when applicable)?

2.3. Are there complete outcome data (80% or above)?

2.4. Is there low withdrawal/drop-out (below 20%)?

**3. Quantitative nonrandomized**

3.1. Are participants (organizations) recruited in a way that minimizes selection bias?

3.2. Are measurements appropriate (clear origin, or validity known, or standard instrument; and absence of contamination between groups

when appropriate) regarding the exposure/intervention and outcomes?

3.3. In the groups being compared (exposed vs. non-exposed; with intervention vs. without; cases vs. controls), are the participants

comparable, or do researchers take into account (control for) the difference between these groups?

3.4. Are there complete outcome data (80% or above), and, when applicable, an acceptable response rate (60% or above), or an acceptable

follow-up rate for cohort studies (depending on the duration of follow-up)?

**4. Quantitative**

**descriptive**

4.1. Is the sampling strategy relevant to address the quantitative research question (quantitative aspect of the mixed methods question)?

4.2. Is the sample representative of the population understudy?

4.3. Are measurements appropriate (clear origin, or validity known, or standard instrument)?

4.4. Is there an acceptable response rate (60% or above)?

**5. Mixed methods** 5.1. Is the mixed methods research design relevant to address the qualitative and quantitative research questions (or objectives), or the

qualitative and quantitative aspects of the mixed methods question (or objective)?

5.2. Is the integration of qualitative and quantitative data (or results*) relevant to address the research question (objective)?

5.3. Is appropriate consideration given to the limitations associated with this integration, e.g., the divergence of qualitative and quantitative

data (or results*) in a triangulation design?

*Criteria for the qualitative component (1.1 to 1.4), and appropriate criteria for the quantitative component (2.1 to 2.4, or 3.1 to 3.4, or 4.1 to 4.4), must be also applied.*

*These two items are not considered as double-barreled items since in mixed methods research, (1) there may be research questions (quantitative research) or research objectives (qualitative research), and (2) data

may be integrated, and/or qualitative findings and quantitative results can be integrated

**Appendix B: SFS Scoring System (Version E)**

**Author: _____________________________**

**Title: __________________________________________________________**

__________________________________________________________

**Source: _____________________________**

**A.PURPOSE (Maximum score = 22)**

1) Is a clear problem statement present?

No Yes

1 2

2) Is a clear statement of the aims of the research made?

(Goal, relevance, why it was thought to be important)

No Yes

1 2

3) Was relevant *background* literature accessed and discussed?

No Yes

1 2

4) Was recent literature accessed and discussed?

>10 years 6-10years 1-5 year period

1 2 3

5) Did the researcher explicitly locate the study within a specific theoretical framework?

No Implied Yes

1 2 3

6) Was a motivation given for use of this framework?

No Yes

1 2

7) Did the article provide theoretical definitions of variables assessed?

No partially/ selectively Yes

1 2 3

8) Did the article provide working definitions of variables assessed?

No partially/ selectively Yes

1 2 3

9) Did the researcher report on a thorough literature study to define the constructs underlying the variables

included in the study?

No Yes

1 2

http://etd.uwc.ac.za 395

**B: METHOLODICAL RIGOUR (Maximum score = 52)**

1) Is the methodology clear and unambiguous?

No Yes

1 2

2) Is the design of the study identified and described in detail?

(How the study will be conducted)

No Yes

1 2

3) Is the research design appropriate to address the aims of the research? (Did the researchers discuss why

they decided to use *quantitative-* RCT, Cohort, Single case, case study, cross sectional etc. OR

*qualitative* - focus group, semi-structured interview etc. OR a *mixed method* research design?)

No Yes

1 2

4) Is the sample described in detail?

No Yes

1 2

5) How was the sample size calculated?

a) For *quantitative* research:

Not reported formula statistical analysis

0 1 2

*****OR*****

b) For *qualitative* research:

Not reported convention saturation

0 1 2

6) Was the recruitment strategy appropriate for the aims of the research?

No Yes

1 2

7) **Data collection**

a) Was the method(s) of data collection described?

No Yes

1 2

b) Was the method appropriate given the research question

No Yes

1 2

8) Theoretical or paradigmatic assumptions/ considerations mentioned

No Yes

1 2

9) Nature of the data required

(i) Will this data support the analysis?

No Yes

1 2

Quantitative:

(ii) Were scales appropriate given the theoretical definition of the variables?

No Yes

1 2

(iii) Were psychometric properties of scales reported?

No Yes

1 2

http://etd.uwc.ac.za 396

********OR********

Qualitative:

(iv) Do the researchers provide adequate information about data collection procedures e.g. gaining access

to the site, field notes, training data gatherers?

No Yes

1 2

(v) Did the researcher comment on his own role, potential bias and influence during data collection?

No Yes

1 2

8) Data analysis

a) Was the analysis described?

No Yes

1 2

b) Was the analysis appropriate given the

i) research question

No Yes

1 2

ii) nature of the data collected

(Did the data support the analysis?)

No Yes

1 2

9) Results

Quantitative:

a) Was statistical significance reported accurately?

No Yes Yes, with alpha levels

1 2 4

******OR******

Qualitative:

b) Was any of the following addressed?

No Yes

Trustworthiness 0 1

Credibility, 0 1

Reflexivity, 0 1

Saturation 0 1

10) Is findings presented clearly and unambiguously?

No Yes

1 2

11) Were findings discussed relevant to the research question and literature reviewed?

No Yes

1 2

2

12) Was a clear conclusion drawn?

No Yes

1 2

13) Did the findings support the conclusion?

No Yes

1 2

14) Were appropriate recommendations made?

No Yes

http://etd.uwc.ac.za 397

1 2

15) Did the authors identify and discuss limitations to the study?

No Yes

1 2

16) Was ethical approval obtained from an identifiable committee/ body?

No Yes

1 2

17) Were participants informed about their rights and responsibilities?

No Yes

1 2

18) Were avenues for recourse identified in the event of questions or concerns?

No Yes

1 2

**C: GENERAL CONSIDERATIONS**

**Quality of information (Maximum score = 5).**

1) Is this study a

Published manuscript Unpublished thesis

2 1

2) Was it

Peer reviewed Externally examined Internally examined only

3 2 1

******************************************************

**Appendix C: PRISMA-P Checklist**

**Table 2| PRISMA-P (preferred reporting items for systematic review and meta-analysis protocols) 2015 checklist: recommended items to address in a systematic review protocol**

| **Section and topic** | **Item No** | **Checklist item** |
| --- | --- | --- |
| **Administrative information** |  |  |
| Title: |  |  |
| Identification | 1a | Identify the report as a protocol of a systematic review |
| Update | 1b | If the protocol is for an update of a previous systematic review, identify as such |
| Registration | 2 | If registered, provide the name of the registry (such as PROSPERO) and registration number |
| Authors: |  |  |
| Contact | 3a | Provide name, institutional affiliation, e-mail address of all protocol authors; provide physical mailing address of corresponding author |
| Contributions | 3b | Describe contributions of protocol authors and identify the guarantor of the review |
| Amendments | 4 | If the protocol represents an amendment of a previously completed or published protocol, identify as such and list changes; otherwise, state plan for documenting important protocol amendments |
| Support: |  |  |
| Sources | 5a | Indicate sources of financial or other support for the review |
| Sponsor | 5b | Provide name for the review funder and/or sponsor |
| Role of sponsor or funder | 5c | Describe roles of funder(s), sponsor(s), and/or institution(s), if any, in developing the protocol |
| **Introduction** |  |  |
| Rationale | 6 | Describe the rationale for the review in the context of what is already known |
| Objectives | 7 | Provide an explicit statement of the question(s) the review will address with reference to participants, interventions, comparators, and outcomes (PICO) |
| **Methods** |  |  |
| Eligibility criteria | 8 | Specify the study characteristics (such as PICO, study design, setting, time frame) and report characteristics (such as years considered, language, publication status) to be used as criteria for eligibility for the review |
| Information sources | 9 | Describe all intended information sources (such as electronic databases, contact with study authors, trial registers or other grey literature sources) with planned dates of coverage |
| Search strategy | 10 | Present draft of search strategy to be used for at least one electronic database, including planned limits, such that it could be repeated |
| Study records: |  |  |
| Data management | 11a | Describe the mechanism(s) that will be used to manage records and data throughout the review |
| Selection process | 11b | State the process that will be used for selecting studies (such as two independent reviewers) through each phase of the review (that is, screening, eligibility and inclusion in meta-analysis) |
| Data collection process | 11c | Describe planned method of extracting data from reports (such as piloting forms, done independently, in duplicate), any processes for obtaining and confirming data from investigators |
| Data items | 12 | List and define all variables for which data will be sought (such as PICO items, funding sources), any pre-planned data assumptions and simplifications |
| Outcomes and prioritization | 13 | List and define all outcomes for which data will be sought, including prioritization of main and additional outcomes, with rationale |
| Risk of bias in individual studies | 14 | Describe anticipated methods for assessing risk of bias of individual studies, including whether this will be done at the outcome or study level, or both; state how this information will be used in data synthesis |

Data synthesis 15a Describe criteria under which study data will be quantitatively synthesised

15b

If data are appropriate for quantitative synthesis, describe planned summary measures, methods of handling data and methods of combining data from studies, including any planned exploration of consistency (such as I2, Kendall’s

τ)

15c Describe any proposed additional analyses (such as sensitivity or subgroup analyses, meta-regression)

Meta-bias(es)

Confidence in cumulative evidence

15d 16

17

If quantitative synthesis is not appropriate, describe the type of summary planned

Specify any planned assessment of meta-bias(es) (such as publication bias across studies, selective reporting within studies)

Describe how the strength of the body of evidence will be assessed (such as GRADE)
